# Peptide-Coated Bacteriorhodopsin-Based Photoelectric Biosensor for Detecting Rheumatoid Arthritis

**DOI:** 10.3390/bios13100929

**Published:** 2023-10-16

**Authors:** Hsiu-Mei Chen, Yi-Hsuan Tsai, Chien-Yi Hsu, Yong-Yi Wang, Cheng-En Hsieh, Jin-Hua Chen, Yu-Sheng Chang, Ching-Yu Lin

**Affiliations:** 1 Department of Chemical Engineering, National Taiwan University of Science and Technology, Taipei 10607, Taiwan; hsiumei@mail.ntust.edu.tw (H.-M.C.); a0189182@gmail.com (Y.-Y.W.); q135135xc@gmail.com (C.-E.H.); 2 Department of Family Medicine, Wan Fang Hospital, Taipei Medical University, Taipei 11696, Taiwan; 106181@w.tmu.edu.tw; 3 Division of Cardiology and Cardiovascular Research Center, Department of Internal Medicine, Taipei Medical University Hospital, Taipei 11031, Taiwan; chienyihsu@tmu.edu.tw; 4 Division of Cardiology, Department of Internal Medicine, School of Medicine, College of Medicine, Taipei Medical University, Taipei 11031, Taiwan; 5 Taipei Heart Institute, Taipei Medical University, Taipei 11031, Taiwan; 6 Graduate Institute of Data Science, College of Management, Taipei Medical University, Taipei 11031, Taiwan; jh_chen@tmu.edu.tw; 7 Office of Data Science, Taipei Medical University, Taipei 11031, Taiwan; 8 Division of Allergy, Immunology and Rheumatology, Department of Internal Medicine, Shuang Ho Hospital, Taipei Medical University, New Taipei City 23561, Taiwan; 9 Division of Allergy, Immunology and Rheumatology, Department of Internal Medicine, School of Medicine, College of Medicine, Taipei Medical University, Taipei 11031, Taiwan; 10 School of Medical Laboratory Science and Biotechnology, College of Medical Science and Technology, Taipei Medical University, Taipei 11031, Taiwan; 11 Ph.D. Program in Medical Biotechnology, College of Medical Science and Technology, Taipei Medical University, Taipei 11031, Taiwan

**Keywords:** photoelectric biosensor, bacteriorhodopsin, peptide, purple membrane, rheumatoid arthritis, diagnosis

## Abstract

An effective early diagnosis is important for rheumatoid arthritis (RA) management. This study reveals a novel RA detection method using bacteriorhodopsin as a photoelectric transducer, a light-driven proton pump in purple membranes (PMs). It was devised by covalently conjugating a PM monolayer-coated electrode with a citrullinated-inter-alpha-trypsin inhibitor heavy chain 3 (ITIH3)^542–556^ peptide that recognizes the serum RA-associated autoantibodies. The direct serum coating decreased the photocurrents in the biosensor, with the reduction in the photocurrent caused by coating with an RA-patient serum that is significantly larger than that with a healthy-control serum (38.1% vs. 20.2%). The difference in the reduction in the photocurrent between those two serum groups widened after the serum-coated biosensor was further labeled with gold nanoparticle (AuNP)-conjugated anti-IgA (anti-IgA-AuNP) (53.6% vs. 30.6%). Both atomic force microscopic (AFM) and Raman analyses confirmed the sequential peptide, serum, and anti-IgA-AuNP coatings on the PM-coated substrates. The reductions in the photocurrent measured in both the serum and anti-IgA-AuNPs coating steps correlated well with the results using commercial enzyme-linked immunosorbent assay kits (Spearman rho = 0.805 and 0.787, respectively), with both a sensitivity and specificity close to 100% in both steps. It was shown that an RA diagnosis can be performed in either a single- or two-step mode using the developed biosensor.

## 1. Introduction

Rheumatoid arthritis (RA) is a chronic, systemic autoimmune disease with a global prevalence rate of approximately 1% [1]. The disease is characterized by inflammation of the synovial joints, which may cause damage to the cartilage and synovial joints if untreated [2]. An early diagnosis and management of RA are a challenge because of the lack of effective diagnostic methods [3]. The presence of two autoantibodies, rheumatoid factor (RF) and an anti-citrullinated protein antibody (ACPA), is associated with the pathogenesis and diagnosis of RA [4]. Immunoglobulin A (IgA)/IgM RFs react with the Fc portion of IgG, with their RA diagnostic sensitivities and specificities respectively ranging from 50–90% and 50–95% [5]. ACPAs are directed against citrullinated proteins, on which positively charged arginine residues are modified into neutral citrulline (Cit) by peptidyl arginine deiminase [6]. RA diagnosis performances of the current commercially available cyclic citrullinated peptide (CCP) assay, as well as its second (CCP2) and third (CCP3) generations, are similar to one another, with respective sensitivities and specificities of 68~79% and 86~96% [4,5]. In addition, a novel serological biomarker, IgG/IgM or IgA anti-citrullinated-inter-alpha-trypsin inhibitor heavy chain 3 (ITIH3)^542–556^ peptide (ITIH3^542–556^ Cit, ALQECit_542_DYIFGNYIECit_556_), was identified from the sera of patients with secondary Sjögren’s syndrome in RA and then used in RA diagnoses with improved sensitivities (80–95%) and specificities (70–100%) [7]. A nanogold-modified electrode coated with this peptide was recently developed as a biosensor using electrochemical impedance spectroscopy, which achieved better sensitivity in the RA diagnosis than the commercial anti-CCP ELISA method [8]. A nanoplasmonic sensing chip coated with ITIH3^542–556^ Cit can be used with a portable solar-powered centrifuge and smartphone reader to detect patients with RA in areas with scarce resources [9].

Different peptide-based biosensors have been developed for disease detection, including acute myocardial infarction, Alzheimer’s disease, diabetes, juvenile idiopathic arthritis, prostate cancer, and RA [10,11,12,13,14,15]. For RA diagnoses, a nanotube-based quartz crystal microbalance biosensor was coated with a 14-mer cyclic citrullinated filaggrin peptide to achieve a 71.9% sensitivity and a 95.8% specificity [16]. A selective and sensitive electrochemical RA biosensor was devised by anchoring a chimeric fibrin−filaggrin synthetic peptide (CFFCP1) on a multiwalled carbon nanotube-polystyrene-based transducer [17]. A multi-epitope peptide-based surface plasmon resonance biosensor detected 90% of RA patient sera without recognizing any healthy ones [10]. The performances of these peptide-based biosensors were comparable to that of a commercial CCP3 assay [8,9]. Recently, RA peptide-based biosensors were devised using the highly selective and sensitive ITIH3^542–556^ Cit peptide as the recognition element [8,9]. These biosensors detect more quickly than an enzyme-linked immunosorbent assay (ELISA) and are easier to use [8,9].

Photoactive bacteriorhodopsin (BR) is the only protein in the purple membrane (PM) of *Halobacterium salinarum*. It functions as a light-driven proton pump to transport protons uni-directionally from the cytoplasmic (CP) to the extracellular (EC) side of PM, so it exhibits a photoelectric effect when connected to an external circuit [18,19,20]. Recently, PM was used as a photoelectric transducer to devise immunosensors for the direct, label-free, quantitative, sensitive, and single-step detection of microorganisms, hemoglobin, and adenosine triphosphate (ATP) [21,22,23,24]. In those studies, uniformly oriented PM was coated on electrodes to conjugate recognition antibodies to capture the targets, and the reduction in the photocurrent generated by the immunosensors was used as the quantification parameter for detection. The reduction in the photocurrent is presumably caused by a light-shielding effect of the bound targets and their restrictive effects on the movement of BR helices that are involved in proton transport [21]. In addition to antibodies, specific nucleic acid aptamers were conjugated to the PM transducer layer to use as recognition elements for diabetes diagnoses and ATP detection [23,24].

This study shows the first peptide-based RA immunosensor that uses PM as a photoelectric transducer and IgA anti-ITIH3^542–556^ Cit peptide as a biomarker. The sensor was prepared by covalently conjugating the ITIH3^542–556^ Cit peptide on a uniformly oriented PM on an electrode. The serum coating resulted in ACPA binding, which caused a reduction in the photocurrent of the sensor. IgA molecules that were captured on the sensor were subsequently labeled with anti-IgA antibodies conjugated with gold nanoparticles (anti-IgA-AuNPs), and a further reduction in the photocurrent was observed. The sera of both RA patients and healthy controls (HCs) were tested to determine a workable reference photocurrent reduction level for RA detection.

## 2. Materials and Methods

### 2.1. Materials

b-PM, a biotinylated form of PM, and oxidized avidin (OA) were prepared as previously described [25]. 3-Aminopropylphosphonic acid (APPA) was obtained from Fluorochem (Derbyshire, UK). Avidin and EZ-Link sulfo-NHS-LC-LC-Biotin were both obtained from Thermo Fisher Scientific (Waltham, MA, USA). A bis-*N*-hydroxylsuccinimide ester that contains two polyethylene glycol (PEG) units, Bis(NHS)PEG2, was obtained from NANOCS (New York, NY, USA). The ITIH3^542–556^ Cit peptide was synthesized by Yao-Hong Biotechnology (New Taipei City, Taiwan). Dressed Gold^®^ goat anti-human IgA (α) conjugates using 40 nm Naked Gold^®^ sols (anti-IgA-AuNPs) were obtained from Bioassay Works (Ijamsville, MD, USA). Graphene oxide (GO) powder and indium tin oxide (ITO) glass (sheet resistance: <15 Ω/sq) were respectively obtained from Graphene Supermarket and Fang Materials (New Taipei City, Taiwan).

### 2.2. Clinical Samples

The serum samples were collected in the Division of Allergy, Immunology, and Rheumatology, Department of Internal Medicine, Shuang Ho Hospital (New Taipei City, Taiwan). Rheumatologists used appropriate classification criteria to diagnose patients with RA. Patients with RA were categorized according to the 2010 American College of Rheumatology (ACR)/European League Against Rheumatism (EULAR) classification criteria [26] or the 1987 ACR classification criteria [27]. This study was approved by the TMU-Joint Institutional Review Board of the study hospital, and all volunteers gave informed consent before being allowed to participate (N201708050, 10 December 2020). The sera were stored at −20 °C until being tested.

### 2.3. Chip Preparation, Characterization, and Serum Detection

Following a previous procedure [22], an ITO glass electrode was sequentially coated with 0.1 mM of APPA, a GO-OA complex linker that was made by mixing GO and OA at a 1:10 weight ratio, and then 1.5 mg/mL of b-PM, which was then subjected to a laminar shear flow (Reynolds number = 0.9). The preparation yielded a b-PM monolayer-coated electrode, which was termed a b-PM chip. To prepare an RA sensor chip, a b-PM chip was first modified with 5 mM Bis(NHS)PEG2 in phosphate buffer (PB) (10 mM, pH 7.4) at room temperature for 30 min; coated with 40 μM of the ITIH3^542–556^ Cit peptide in phosphate-buffered saline (PBS) (0.14 M, pH 7.4) at 4 °C for 2 h; and finally, blocked with 0.2 M of glycine in PB. For detection, the RA sensor chip was drop-coated with 5 µL of the 100-fold diluted serum in PBS at 4 °C for 2 h and briefly rinsed with PBS, and the photocurrent was measured. For signal enhancement, the serum-coated RA sensor chip was further drop-coated with 5 µL of 20 pM (particle molarity) anti-IgA-AuNPs in PBS at 4 °C for 2 h. After a brief rinse with PBS, the photocurrent was measured again.

Atomic force microscopy (AFM), photocurrent measurement, and Raman spectroscopy were conducted as described previously [22]. To collect the photocurrent, the chip comprised of b-PM was irradiated with an 80 mW green continuous wave laser at an on–off pattern to produce a pair of two transient photocurrent spikes with opposite polarities. The total photocurrent density of the chip was defined as the difference between the maximum light-on and the minimum light-off photocurrent densities. The photocurrent reduction level was defined as the reduction percentage in the total photocurrent density of the RA sensor chip after incubation with a serum or anti-IgA-AuNP solution by taking the total photocurrent density of another control chip incubated in PBS as the reference.

## 3. Results and Discussion

### 3.1. Fabrication, Characterization, and Detection

**Figure 1:**
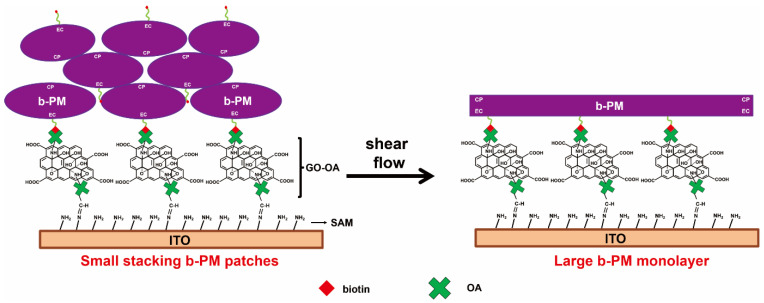
Schematic illustration of the structure and the preparation of a b-PM monolayer-coated ITO (figure adapted from [22]).

b-PM, with PM conjugated with biotin on its EC side, was utilized to prepare a b-PM monolayer-coated electrode that was used as the photoelectric transducer for sensing. The right half of Figure 1 depicts the structure of the transducer. For preparation, b-PM was first drop-coated on a self-assembled monolayer (SAM)-coated ITO electrode using a GO-OA complex as the linker (Figure 1, left half). The SAM layer was formed by APPA through its phosphonate headgroup. As evidenced by the AFM and Fourier transform infrared spectroscopy (FTIR) analyses in our previous study [22], the GO-OA complex linker has a double-sided planar structure with OA granules uniformly deposited on either side of each GO sheet via the covalent linkages between the amines of OA and the epoxides of the GO basal planes. The complex linker was bound to the SAM-coated ITO via Schiff’s base linkages between the surface amines on ITO and the aldehydes of OA that were positioned on the lower basal plane of GO. b-PM was unidirectionally immobilized over the linker due to the strong affinity between the biotin moiety on the EC side of b-PM and the OA conjugated onto the upper basal plane of the linker. However, due to the hydrophilicity of the linker as well as the ionic interaction between the counter-oriented b-PM patches, the drop-coated b-PM was originally stacked in multilayers as conceptually illustrated in the right half of Figure 1. To mobilize and disintegrate the upper stacking b-PM layers and to fill up the whole substrate surface, a post-deposition washing procedure was first introduced in our previous study [22]. With the merits of the intrinsic fluid nature of cellular membranes and of the surfactant characteristic of GO sheets, the dynamic transition, redistribution, and fusion of b-PM patches occurred, resulting in the formation of a large, continuous, unidirectional, and single monolayer of b-PM covering the substrate, which also benefits, and hence, optimizes the subsequent conjugation of the recognition peptide. Without preformation of this b-PM monolayer, most of the peptides might not be directly conjugated on the foundation b-PM layer but on the upper stacking b-PM layers instead, which might possibly be washed away during the subsequent coating and washing steps. Therefore, the GO-OA linker and the post-deposition washing procedure were constantly applied in our current and previous PM-based-sensor studies [22,23,24].

**Figure 2:**
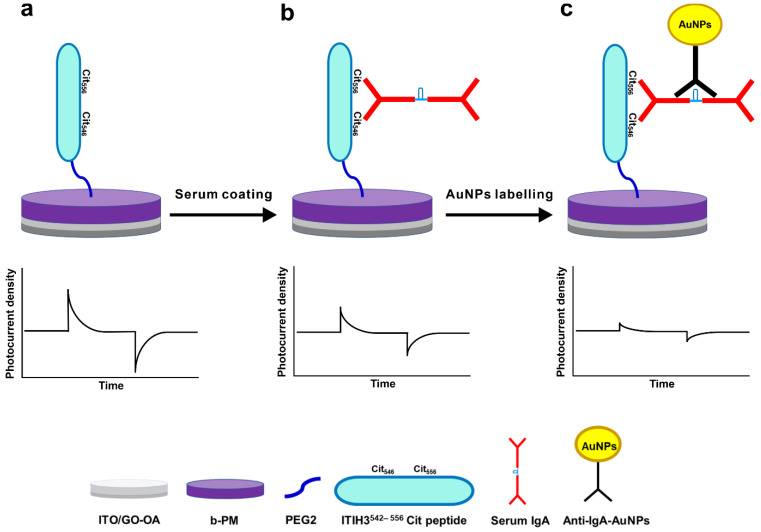
Schematic (upper) structures and (lower) photocurrent responses of (**a**) a rheumatoid arthritis (RA) sensor chip, (**b**) a serum-IgA-coated RA chip, and (**c**) a serum-IgA-coated RA chip further labeled with anti-IgA-gold nanoparticles (AuNPs). Although IgA, IgG, and IgM were all possibly recognized by the peptide, (**b**) shows only the binding of IgA to reduce the complexity of the depiction.

Figure 2a shows the schematics and the sensing mechanism of the RA sensor chip. To covalently conjugate the recognition ITIH3^542–556^ Cit peptide on the b-PM monolayer-coated chip, we employed Bis(NHS)PEG2, a homobifunctional amine-reactive linker containing N-hydroxysuccinimide (NHS) ester groups at either side of the two tandem PEG groups (PEG2). The two terminal NHS groups reacted at the amine ends of the peptide and the Lys159 residue, which is the only lysine exposed on the CP side of the immobilized b-PM (PDB: 4Y9H), respectively resulting in an RA sensor chip featuring a PEG2 spacer between them.

For detection, a serum sample was directly coated onto the RA sensor chip, resulting in the deposition of antibodies that were recognized by the ITIH3^542–556^ Cit peptide immobilized on the sensor top. According to a previous study [7], IgA, IgG, and IgM were all possibly recognized by the peptide, with IgA exhibiting the most significant binding for an RA diagnosis. Therefore, Figure 2b depicts only the adsorption of IgA on the RA sensor chip. Due to the colossal size and molecular weight of antibodies, the binding of sera would cause a reduction in the photocurrent generation of the sensor chip, as depicted in the schematic photocurrent responses of Figure 2a,b.

To identify the captured IgA and enhance the detection signal, the serum-coated chip was further labeled with 40 nm AuNPs conjugated with anti-IgA IgG, i.e., anti-IgA-AuNPs, as illustrated in Figure 2c. The information provided by the supplier showed that the 40 nm AuNPs exhibited a maximum absorbance peak at around 530 nm, which was close to the wavelength (532 nm) of the green laser we used to irradiate the sensor chip. Therefore, the binding of anti-IgA-AuNPs presumably would further block part of the incident light, and hence, decrease the photocurrent production of the sensor chip, as depicted in the lower panels of Figure 2b,c.

**Figure 3:**
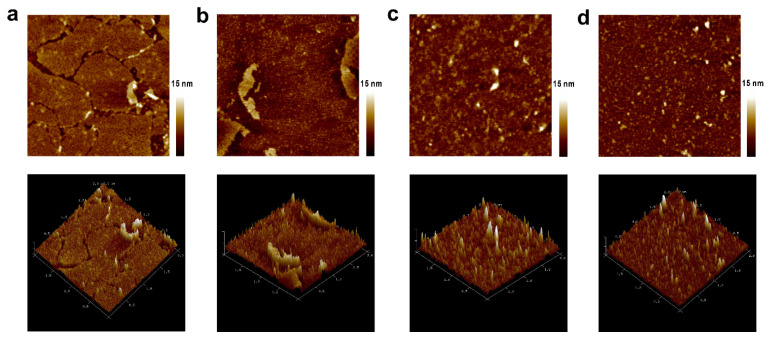
AFM (upper) topographic and (lower) 3D images of different layer-by-layer fabricated mica. The topmost layer in each image is (**a**) biotinylated purple membrane (b-PM), (**b**) ITIH3^542–556^ Cit peptide, (**c**) healthy control (HC) serum, and (**d**) anti-IgA-gold nanoparticles (AuNPs). Scan size: 2 µm.

As shown in the AFM images of Figure 3a, the foundational b-PM layer was mainly composed of small, contiguous, irregularly shaped monolayers whose borders were partially connected. All small monolayers exhibited a cracked interior morphology, so they were all uniformly oriented. According to a previous study, the cracked morphology represents the cytoplasmic side of PM [28]. The topographic morphology of the peptide-coated surface (Figure 3b) had a very different texture from that of the b-PM layer. A large, continuous, fused layer was observed with many narrow, needle-like structures standing on the top, which evidenced the coating of the ITIH3^542–556^ Cit peptide onto the b-PM foundational layer. Subsequently, the size of the materials deposited on the topmost layer first increased after the serum coating and then decreased after the anti-IgA-AuNP coating, respectively implying the attachment of antibodies and AuNPs.

**Figure 4:**
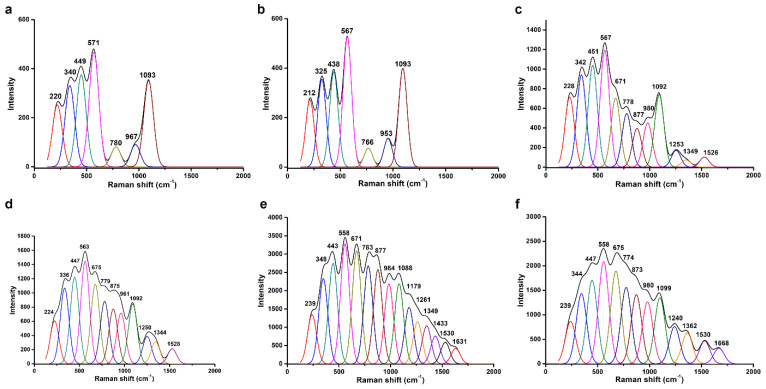
Raman spectra for ITO electrodes that were fabricated using (**a**) 3-aminopropylphosphonic acid (APPA), (**b**) graphene oxide (GO)-oxidized avidin (OA) complex linker, (**c**) biotinylated purple membrane (b-PM), (**d**) ITIH3^542–556^ Cit peptide, (**e**) healthy control (HC) serum, and (**f**) anti-IgA-gold nanoparticles (AuNPs) at the top. The bands in each spectrum were identified using the PeakFit deconvolution program.

Figure 4 shows the Raman spectra of ITO electrodes fabricated with different topmost layers. The bands of each deconvoluted spectrum were assigned and are listed in Table 1, according to previous studies. The spectra of substrates topped with APPA and with GO-OA were similar, possibly due to the scanty coating amount of GO-OA. The b-PM coating resulted in the appearance of several additional bands at 671, 778, 877, 1253, 1349, and 1526 cm^−1^ caused by the amide III, C=C stretching, C-S stretching, tryptophan (Trp), and tyrosine (Tyr) of BR. The relative intensities of those additional bands compared to those of the characteristic bands of ITO at 563~567 and 1092 cm^−1^ became stronger after conjugation with the ITIH3^542–556^ Cit peptide. Subsequent coating with the human serum yielded two additional minor bands at 1179 and 1433 cm^−1^, which nevertheless were not observed in the electrode’s spectrum further coated with anti-IgA-AuNPs. The distinction among the spectra of those electrodes topped with different molecules evidenced the sequential layer-by-layer coating of the b-PM, peptides, human serum, and labeling antibodies on the GO-OA-coated electrode. The surface-enhanced Raman scattering effect of AuNPs was not evident in Figure 4f, suggesting that the anti-IgA-AuNPs may be scarcely coated so that AuNPs were separately distributed on the electrode surface.

**Figure 5:**
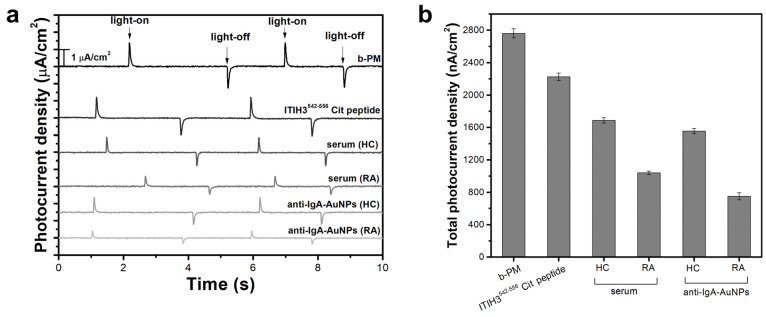
(**a**) Typical photocurrent responses and (**b**) total photocurrent densities of chips coated with different top layers during the sequential layer-by-layer fabrication. The data are presented as the average value for three chips of a single type with one standard deviation (RSD < 5%).

The results of the photocurrent measurements also confirmed the sequential layer-by-layer coating of different materials on the GO-OA-coated electrode. As shown in Figure S1, Supplementary Materials, the b-PM monolayer-coated transducer electrode generated the greatest photocurrent at pH 8.5 so all measurements were conducted at pH 8.5. The chip also displayed extraordinary measurement repeatability and stability under repeated irradiations (RSD = 0.6%, Figure S2). As shown in Figure 5a, the pristine b-PM-coated electrode generated the greatest photocurrent intensity, with a photocurrent profile typically comprising a pair of two transient spikes with opposite polarity in response to the on–off status of the exciting laser. Peak intensities of the b-PM-coated chip gradually decayed along with the sequential coating of the ITIH3^542–556^ Cit peptides, human serum, and anti-IgA-AuNPs. Total photocurrent densities of those different top-layered b-PM chips are shown in Figure 5b. After peptide conjugation, the photocurrent was significantly reduced by 19.5% ± 1.9%. Since none of the 20 naturally occurring amino acids adsorb visible radiation, the photocurrent reduction was attributed to factors other than the illumination intensity. It was previously summarized that during light-driven proton transportation, the CP side of helices F and G of BR swings open to form a narrow channel to allow a proton taken from this side [29]. In the current study, the ITIH3^542–556^ Cit peptide was possibly conjugated to the Lys159 residue that is located on the C-terminal end of helix E, followed closely by the N-terminal end of helix F. Therefore, peptide conjugation can likely restrain the movement of helix F on immobilized BR, hampering the proton uptake and consequently decreasing the photocurrent generation.

Figure 5b also clearly shows that the subsequent serum coating caused a further reduction in the generated total photocurrent density, as coating with an RA-serum sample led to a significantly greater reduction than the coating with the HC serum (53.3% ± 2.6% vs. 24.1% ± 2.3%). The photocurrent reduction resulting from the serum coating indicated that the ITIH3^542–556^ Cit peptides conjugated onto the immobilized b-PM surface maintained their binding activity toward ACPAs. The significant difference in the reduced levels between those two different serum samples suggested that the as-prepared RA sensor was sufficiently sensitive to directly distinguish the extents of citrullination between the antibodies in the healthy controls and patient sera with a single-step analysis.

Differences in the photocurrent reduction levels were even more profound after the serum-coated chips were further labeled with anti-IgA-AuNPs. After AuNP labeling, the total photocurrent density of the HC-serum-coated chip was only slightly reduced (7.9% ± 2.8%), while a 27.7% ± 4.7% photocurrent reduction was achieved with the RA-serum-coated chip. These results imply that the IgA amount of ACPAs in the RA serum was much greater than that in the HC serum, which agrees with previous findings that the concentration of IgA autoantibodies is significantly higher in RA patients [7]. Therefore, the two-step analysis with the as-prepared RA sensor, with an initial serum coating followed by anti-IgA-AuNP labeling, provides a feasible method for RA detection.

The long-term stability of the as-prepared RA sensor was investigated monthly. The sensor was stored in 10 mM of PB containing 100 mM of NaCl at pH 8.0 and 4 °C. The results showed that the RA sensor chip maintained its full activity toward ACPAs in the RA serum for 3 months. Starting from the 4th month, the peptide-coated chip generated 7.6% ± 5.0% more photocurrents than it had generated beforehand, and this photocurrent production level remained thereafter. Meanwhile, its photocurrent reduction level on the coating with the RA serum fell by 11~13%. Detachment of the ITIH3^542–556^ Cit peptides from the b-PM-coated electrode surface possibly occurred during long-term storage, resulting in the increase in the photocurrent production and losing the ACPA-binding ability of the sensor chip.

### 3.2. Clinical Samples

**Figure 6:**
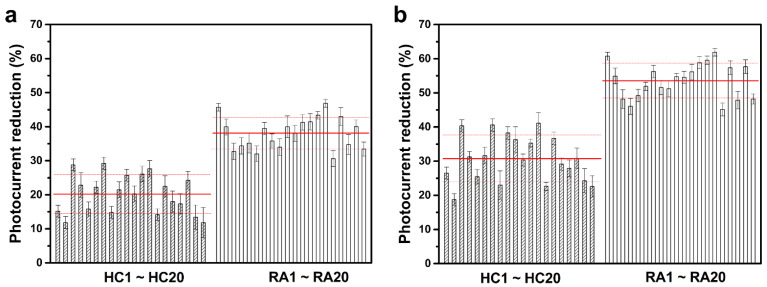
(**a**) Reductions in photocurrent of rheumatoid arthritis (RA) sensors after coating with various serum samples. (**b**) Reductions in the photocurrent of various serum-coated RA sensors after further labeling with anti-IgA-gold nanoparticles (AuNPs). All data are presented as the average value for three chips of a single type with one standard deviation. Solid and dashed red lines respectively indicate the average and one standard deviation of the reduction percentages in each tested sample group.

For the clinical test, 40 female serum samples from 20 RA patients (51.10 ± 13.46 years old) and 20 age-matched HCs (50.80 ± 13.01 years old) were collected by medical professionals, and each was analyzed using the developed RA sensor. Table 2 shows clinical and demographic characteristics of individuals who contributed serum samples to this study. Figure 6 shows the analytical results of the sensor study on each individual serum sample. As shown in Figure 6a, in the first direct detection step where the serum samples were directly coated on the RA sensor chips, the average of the photocurrent reduction resulting from the coating with the RA-serum samples was 38.1% ± 4.7%, which was significantly greater than the average value caused by the coating with the HC-serum samples (20.2% ± 5.7%). The analytical results between the HC- and RA-serum samples were more distinct in the second signal enhancement step, where the serum-coated chips were further labeled with anti-IgA-AuNPs. As shown in Figure 6b, after labeling, the HC-serum coated chips yielded an average further photocurrent reduction of 30.6% ± 6.8%, while the RA-serum coated ones had an average reduction of 53.6% ± 5.0%. The higher photocurrent reductions caused by labeling were higher with the RA-serum samples than with the HC-serum samples.

**Figure 7:**
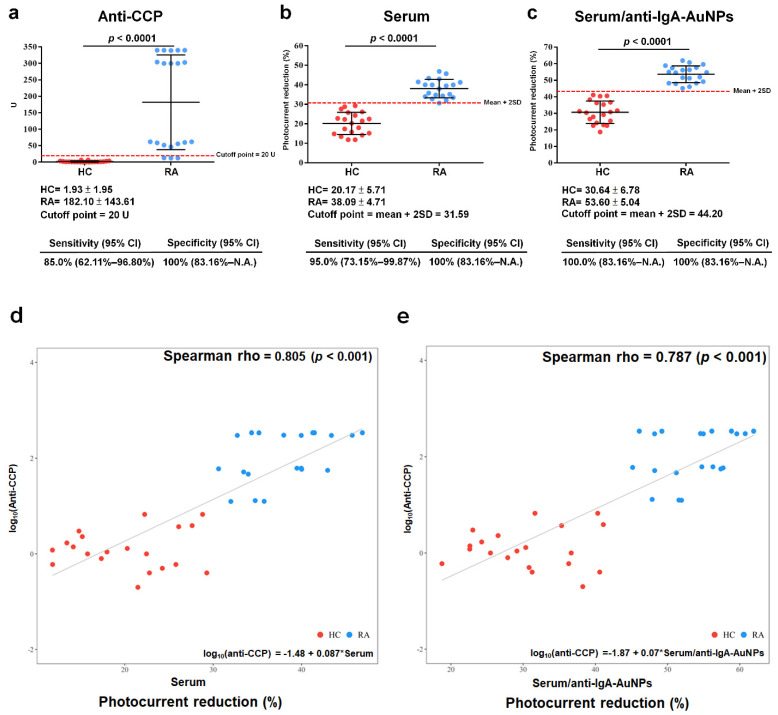
Statistical analysis was conducted with rheumatoid arthritis (RA) patients and healthy controls (HCs). (**a**) An anti-cyclic citrullinated peptide (CCP) ELISA kit (cutoff point = 20 U), (**b**) RA sensor chip coated with serum samples (mean ± 2 standard deviations (SDs) = 31.59%), and (**c**) RA sensor chip after coating with serum samples and further labeling with anti-IgA-gold nanoparticles (AuNPs; serum/anti-IgA-AuNPs, mean ± 2 SDs = 44.20%). Correlation of the anti-CCP ELISA kit with (**d**) RA sensor chip coated with serum samples and (**e**) RA sensor chip coated with serum/anti-IgA-AuNPs. The red dot denoted HC, while the blue dot denoted RA. N.A., not available.

In the anti-CCP ELISA test, the average autoantibody concentration of the RA samples was 182.10 ± 143.61 U, and the average autoantibody concentration of the HC samples was 1.93 ± 1.93 U, as determined via statistical analysis of the data depicted in Figure 7a. According to the vendor’s recommendation, the proposed cutoff point was set to 20 U, resulting in a sensitivity of 85% (62.11–96.80%) and a specificity of 100% (83.6%~NA). NA stands for “not available”. The serum samples were coated onto the RA sensor chips, as seen in the statistical analysis of Figure 7b. The average photocurrent reduction from coating the RA-serum samples was 38.09% ± 4.71%, while the average photocurrent reduction from coating the HC-serum samples was 20.17% ± 5.71%. The suggested cutoff point was established as the mean ± two SDs, (31.59%), leading to an elevated sensitivity of 95.0% (73.15–99.87%) and a specificity of 100% (83.16%–NA). According to the statistical analysis in Figure 7c, the serum samples were coated onto the RA sensor chips and then labeled with anti-IgA-AuNPs (serum/anti-IgA-AuNPs). The average photocurrent reduction from coating the RA-serum samples was found to be 53.60% ± 5.04%, whereas the average photocurrent reduction from coating the HC-serum samples was observed to be 30.64% ± 6.78%. The suggested cutoff point was established at the mean ± 2SDs (44.20%), resulting in a higher sensitivity of 100.0% (83.16%–NA) and a specificity of 100% (83.16%–NA). A nonlinear regression was used to assess the autoantibody measurements. Relationships between anti-CCP ELISA and the RA sensor chips coated with the serum samples or the serum/anti-IgA-AuNPs were estimated using Spearman’s correlations. Statistically significant positive correlations were identified: log10 (anti-CCP) versus the RA sensor chip coated with the serum samples (rho = 0.805, *p* < 0.001) and log10 (anti-CCP) versus the RA sensor chip coated with the serum/anti-IgA-AuNPs (rho = 0.787, *p* < 0.001), as shown in Figure 7d,e, respectively. An RA sensor chip had a better diagnostic performance than the anti-CCP ELISA kit, which increased the sensitivity at a fixed 100% specificity. The RA sensor chips coated with the serum/anti-IgA-AuNPs exhibited heightened sensitivity compared to the serum-coated chips. A statistically significant positive correlation existed between the RA sensor chips and anti-CCP ELISA kits in diagnosing RA.

According to the previous study using IgA anti-ITIH3542-556 Cit ELISA to measure RA, the best detection results were obtained when the serum was diluted 100-fold [7]. This study investigated the effect of the serum dilution factors, including 100-fold, 500-fold, 1000-fold, 1500-fold, and 2000-fold, on the diagnosis performance of the as-prepared RA sensor chip. As shown in Figure S3a, when the RA sensor chip was first coated with the serum, the difference in the photocurrent reduction levels between the RA and HC serum groups shows a linear dynamic range between the 100-fold and 1500-fold dilution of the serum. However, in the subsequent labeling with anti-IgA-AuNPs, only the 100-fold dilution resulted in a substantial difference in the photocurrent reduction levels between the RA and HC serum groups (Figure S3b). The difference in the photocurrent reduction levels between these two serum groups drops significantly at the 500-fold dilution and stays low at further dilutions, implying that the amount of human IgA captured by the RA sensor chip from the ≥500-fold diluted serum was very scarce and insufficient for further anti-IgA-AuNP labeling. Villa Mde et al. reported that the linear range of a chimeric fibrin−filaggrin synthetic peptide-coated amperometric immunosensor for diagnosing RA is a 100- to 200-fold serum dilution, without reporting the detection limit [17]. Drouvalakis et al. found that the linear range of a CCP-coated nanotube-based biosensor for RA detection is a 200- to 500-fold serum dilution, also without reporting the detection limit [16]. Lin et al. showed that the linear range of an ITIH3^542–556^ Cit peptide-coated nanotube-based biosensor for RA detection is a 2- to 64-fold serum dilution, but they also did not disclose the detection limit [9]. Prak et al. demonstrated that a nanoplasmonic sensing chip coated with ITIH3^542–556^ Cit may detect RA over a linear range of a 2- to 16-fold serum dilution, and the detection limit of the biosensor is unknown [9]. Chinnadayyala and Cho found that the linear range of an electrochemical impedimetric immunosensor with CCP for the early detection of RA is 0.1 to 800 IU/mL, and the detection limit of the biosensor is 0.6 IU/mL [30]. In this study, the detection limit of the RA sensor chip has not been investigated yet. Liao et al. reported that the performance of the ITIH3^542–556^ Cit peptide for detecting RA using an ELISA had a sensitivity of 94.0–100% and a specificity of 79.6–98.4%, respectively [7]. Drouvalakis et al. showed that CCP-coated nanotube-based biosensors to diagnose RA had a 71.9% sensitivity and a 95.8% specificity [16]. Szarka et al. found that the ProteOn^TM^ GLH sensor chip with the multi-epitope citrulline-peptide in surface plasmon resonance analysis can detect RA with a 90% sensitivity and a 100% specificity [10]. As shown in Figure 7a–c, the current RA sensor chip exhibits the same 100% specificity as the anti-CCP kit both in the detection step of the serum coating and in the subsequent labeling step of anti-IgA-AuNP coating. However, the sensitivity of the RA sensor chip was much better than that of the anti-CCP kit (85%), with a sensitivity of 95% and 100% in the detection step of the serum coating and in the subsequent labeling step of anti-IgA-AuNP coating, respectively.

**Table 1:** Raman-band (cm^−1^) assignment for ITO electrodes fabricated with different topmost layers ^a^.

APPA	GO-OA	b-PM	Peptide	HC Serum	Anti-IgA-AuNPs	Band Assignment	References
220	212	228	224	239	239	ITO	-
340	325	342	336	348	344	ITO	-
449	438	451	447	443	447	ITO	-
571	567	567	563	558	558	ITO	-
		671	675	671	675	C-S stretching/Tyr	[31,32]
780	766	778	779	783	774	ITO/Trp	[31,32,33]
		877	875	877	873	Tyr	[31,32,33]
967	953	980	961	984	980	ITO	-
1093	1093	1092	1092	1088	1099	ITO	-
				1179		Trp/Phe	[31]
		1253	1250	1261	1240	Amide III/retinal	[34,35]
		1349	1344	1349	1362	Amide III/retinal	[34,35]
				1433		CH_2_ deformation	[34]
		1526	1528	1530	1530	C=C stretching, retinal	[35]
				1631	1668	Trp/Phe	[31]

^a^ Data originated from the deconvoluted Raman spectra derived from Figure 4. APPA, 3-aminopropylphosphonic acid; GO-OA, graphene oxide-oxidized avidin; b-PM, biotinylated purple membrane; HC, healthy control; AuNPs, gold nanoparticles; Tyr, tyrosine; Trp, tryptophan; Phe, phenylalanine.

**Table 2:** Demographic and clinical characteristics of individual subjects contributing serum for healthy controls (HCs) and rheumatoid arthritis (RA) patients.

Characteristic ^a^	HC ^b^	RA ^c^
	*n* = 20	*n* = 20
Age (years)	51.10 ± 13.46	50.80 ± 13.01
Gender		
Female	20	20
Disease duration (median in years)	N.A. ^c^	5.4 ± 6.41
DAS 28	N.A.	4.4 ± 1.67
Clinical tests		
RF-positive (%)	0	94.7
Anti-CCP-positive (%)	0	85.0
CRP-positive (%)	N.A.	35.0
ESR-positive (%)	N.A.	90.0

^a^ DAS 28, disease activity score 28; RF, rheumatoid factor; Anti-CCP, anti-cyclic citrullinated peptide antibody; CRP, C-reactive protein; ESR, erythrocyte sedimentation rate. ^b^ HCs were tested by Union Clinical Laboratory in Taiwan: Rheumatoid factor (Latex RF reagent, Siemens Medical Solutions Diagnostics, USA; cutoff < 15.0 IU/mL); INOVA QUANTA Lite CCP3 IgG ELISA (INOVA DIAGNOSTICS; cutoff < 20 U). ^c^ Patient samples were analyzed at the Department of Laboratory Medicine, Shuang Ho Hospital, Taipei Medical University, Taiwan: Rheumatoid factor (Latex RF reagent, Siemens Medical Solutions Diagnostics; cutoff < 15.0 IU/mL), “DIESSE” CHORUS ANTI-CCP (DIESSE DIAGNOSTICA SENESE; cutoff < 7.0 AU/mL), CRP (CardioPhase^®^ hsCRP, SIEMENS, Munich, Germany; cutoff of <0.5 mg/dL), and ESR 1 h (VACUETTE^®^, Greiner Bio-One, Kremsmünster, Austria; cutoff < 10 mm/h). N.A., not available.

## 4. Conclusions

In this study, we developed a peptide-based RA sensor chip for autoantibody detection in RA diagnostics. The diagnostic performance of the RA sensor chip was superior to that of the current gold standard, an anti-CCP ELISA kit. We determined diagnostic cutoff values for both the RA sensor chip coated with the serum and the serum/anti-IgA-AuNPs. The proposed RA sensor chip can be used with anti-CCP ELISA kits in the clinic. In RA detection, the RA sensor chips have a specificity of 100% in both detection methods. The sensitivity of these sensor chips, when used with the serum alone or with the serum combined with anti-IgA-AuNPs, is 95% and 100%, respectively. Although the subsequent labeling with anti-IgA-AuNPs increases by 5% in sensitivity, the single-step detection with the serum alone may be considered a more cost-effective option. For practical applications, it is imperative to obtain substantial amounts of serum samples to validate the clinical performance of both detection methods. In addition, equipment miniaturization and parallel multiple assays will benefit the commercialization of this RA sensor.

## Data Availability

Not applicable.

## Supplementary Materials

The following supporting information can be downloaded at: https://www.mdpi.com/article/10.3390/bios13100929/s1, Figure S1: Effect of pH on the relative total photocurrent density of the pristine b-PM chip; Figure S2: Effect of irradiation cycles on the relative total photocurrent density of the pristine b-PM chip; Figure S3: Effect of the dilution factor of serum on the difference in the photocurrent reduction levels between the RA and HC serum groups.
