# Supplementary material for: Peptide-Coated Bacteriorhodopsin-Based Photoelectric Biosensor for Detecting Rheumatoid Arthritis
Source: Biosensors (Basel). 2023 Oct 16;13(10):929. doi: 10.3390/bios13100929 (PMC10605345; doi:10.3390/bios13100929)
Supplement: Supplementary file 1 [file biosensors-13-00929-s001.zip › biosensors-2579172-supplementary.pdf]

biosensors

MDPI

Supplementary Materials

Peptide-Coated Bacteriorhodopsin-Based Photoelectric Biosensor for Detecting Rheumatoid Arthritis

Hsiu-Mei Chen [1], Yi-Hsuan Tsai [2,†], Chien-Yi Hsu [3,4,5,†], Yong-Yi Wang [1], Cheng-En Hsieh [1], Jin-Hua Chen [6,7], Yu-Sheng Chang [8,9,*] and Ching-Yu Lin [10,11,*]

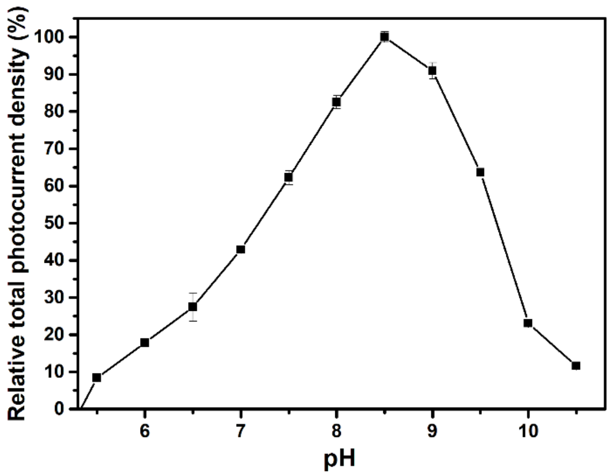

Figure S1. Effect of pH on the relative total photocurrent density of the pristine b-PM chip. The value of the total photocurrent density generated at pH 8.5 was taken as 100%. Data are presented as the average value for three chips at the same pH with one standard deviation.

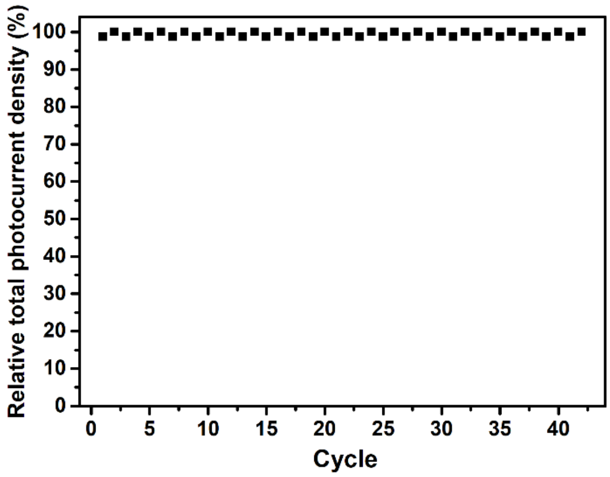

Figure S2. Effect of irradiation cycles on the relative total photocurrent density of the pristine b-PM chip. The value of the total photocurrent density generated at the first cycle was taken as 100%. Data are presented as the average value for three chips with one standard deviation.

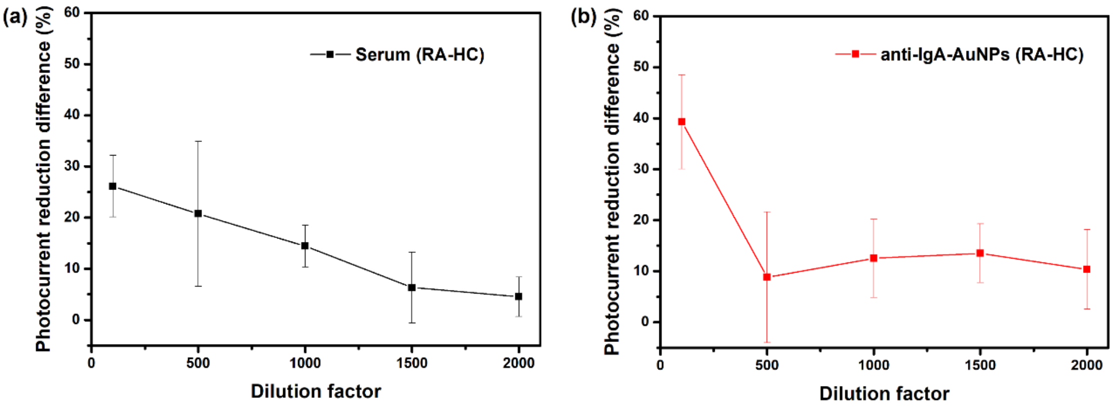

Figure S3. Effect of the dilution factor of serum on the difference in the photocurrent reduction levels between the RA and HC serum groups. Data are presented as the average value for three chips with one standard deviation.
